# Supplementary material for: Assessing the Need for Multiplex and Multifunctional Tick-Borne Disease Test in Routine Clinical Laboratory Samples from Lyme Disease and Febrile Patients with a History of a Tick Bite
Source: Trop Med Infect Dis. 2021 Mar 17;6(1):38. doi: 10.3390/tropicalmed6010038 (PMC8005980; doi:10.3390/tropicalmed6010038)
Supplement: Supplementary file 1 [file tropicalmed-06-00038-s001.pdf]

Supplementary material

Assessing the Need for Multiplex and Multifunctional Tick-Borne Disease Test in Routine Clinical Laboratory Samples from Lyme Disease and Febrile Patients with a History of a Tick Bite

Kunal Garg [1*], T. Sakari Jokiranta [2], Sanna Filén [2] and Leona Gilbert [1,*]

[1] Tezted Ltd, Mattilaniemi 6-8, 40100, Jyväskylä, Finland.
[2] United Medix Laboratories, Kivihaantie 7, 00310, Helsinki, Finland.
* Corresponding Authors: kunal.garg@tezted.com; leona.k.gilbert@tezted.com

	Specimen group	Fisher test type	*Borrelia* spirochete species	*Borrelia* persistent forms	*Babesia microti*	*Bartonella henselae*	*Ehrlichia chaffeensis*	*Rickettsia akari*	Coxsackievirus	Epstein-Barr virus	Human parvovirus B19	*Mycoplasma* species
**IgM**	Lyme disease<br>Febrile	Two tailed *p* value	0.38	1	1	1	0.22	1	0.22	0.05	0.19	1
**IgG**	Lyme disease<br>Febrile	Two tailed *p* value	0.02	0.48	0.76	0.37	1	0.59	1	0.53	1	0.48

Figure S1. Statistical association or dependence was observed between Lyme disease and febrile patient group's IgM and IgG responses to Epstein-Barr virus and Borrelia spirochete species, respectively. Borrelia spirochete species and Borrelia persistent forms refer to *Borrelia burgdorferi sensu stricto, Borrelia afzelii,* and *Borrelia garinii* in spirochete and persistent form, respectively. Similarly, Mycoplasma species refers to *Mycoplasma fermentans* and *Mycoplasma pneumoniae.* The *p*-value originates from Fisher's exact test that was used to assess the statistical differences in IgM or IgG immune responses between LD (positive and negative) and febrile patient groups. The two-tailed *p* values for Fisher's exact test was calculated using the GraphPad (https://www.graphpad.com/quickcalcs/contingency1/). Fisher's exact test results with *p* values < 0.05 were considered statistically associated or dependent.

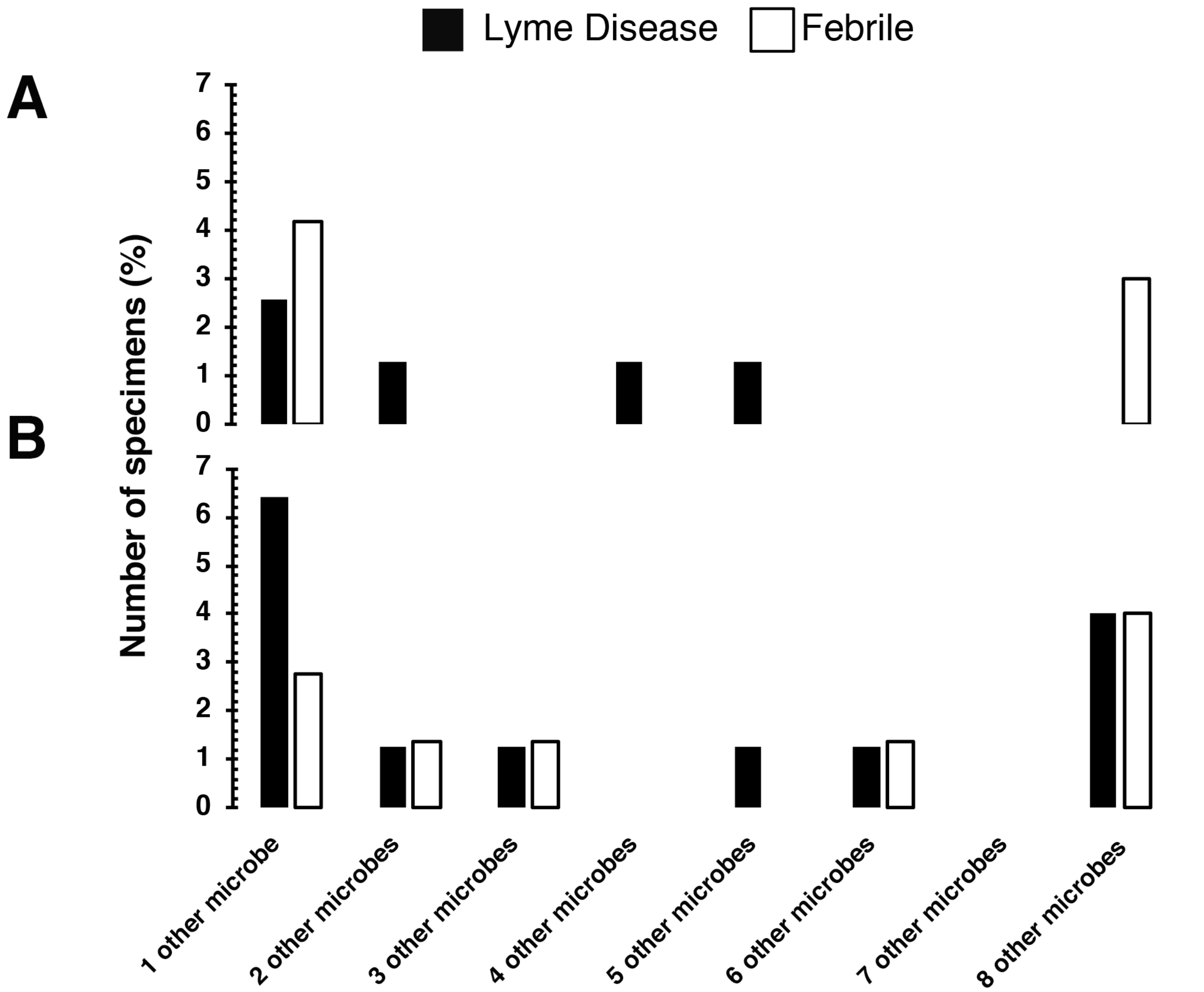

Figure S2. Lyme disease and febrile patient specimens demonstrated (A) IgM and (B) IgG immune responses for up to eight other microbes with Borrelia using TICKPLEX® test. In the present figure, other microbes refer to *Babesia microti*, *Bartonella henselae*, *Ehrlichia chaffeensis*, *Rickettsia akari*, Coxsackievirus, Epstein-Barr virus, Human parvovirus B19, *Mycoplasma fermentans* and *Mycoplasma pneumoniae* on the index test.

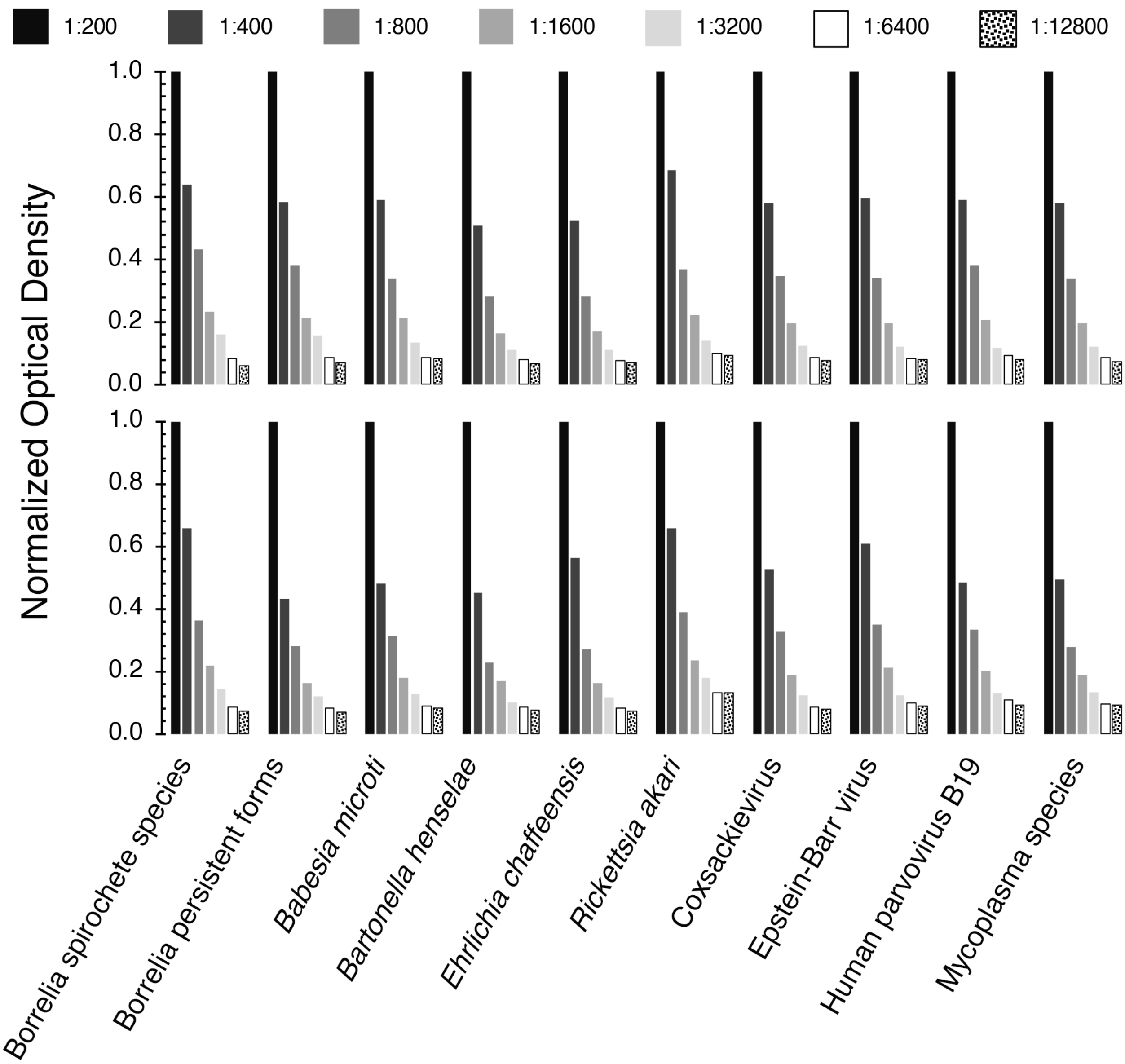

Figure S3. No (A) IgM or (B) IgG unspecific binding is observed on TICKPLEX® test. Borrelia spirochete species and Borrelia persistent forms refer to *Borrelia burgdorferi sensu stricto, Borrelia afzelii,* and *Borrelia garinii* in spirochete and persistent form, respectively. Similarly, Mycoplasma species refers to *Mycoplasma fermentans* and *Mycoplasma pneumoniae.*

Table S1. Normalized IgM optical density values for Lyme disease positive (sera ID 1-48), negative (sera ID 49-78), and febrile patients (sera ID 79-150) from index test.

Sera ID	Borrelia spirochete species	Borrelia persistent forms	*Babesia microti*	*Bartonella henselae*	*Ehrlichia chaffeensis*	*Rickettsia akari*	Coxsackievirus	Epstein-Barr virus	Human parvovirus B19	Mycoplasma species
1	0.485	0.288	0.360	0.359	0.224	0.354	0.201	0.216	0.306	0.368
2	1.065	0.850	0.769	0.869	0.618	0.999	0.503	0.491	0.722	0.736
3	0.647	0.496	0.505	0.486	0.326	0.512	0.289	0.279	0.432	0.529
4	0.366	0.328	0.360	0.395	0.258	0.443	0.214	0.257	0.342	0.374
5	0.385	0.332	0.387	0.395	0.246	0.417	0.208	0.245	0.342	0.368
6	0.342	0.297	0.323	0.365	0.224	0.386	0.195	0.212	0.311	0.351
7	0.361	0.336	0.414	0.444	0.273	0.424	0.346	0.242	0.395	0.379
8	0.504	0.394	0.436	0.511	0.277	0.468	0.252	0.260	0.390	0.443
9	1.289	0.916	0.887	0.924	0.584	0.936	0.557	0.506	0.859	0.822
10	0.495	0.491	0.618	0.778	0.493	0.810	0.415	0.465	0.606	0.603
11	0.371	0.310	0.360	0.389	0.262	0.411	0.204	0.223	0.316	0.368
12	0.404	0.367	0.317	0.346	0.228	0.380	0.204	0.242	0.327	0.339
13	0.547	0.429	0.446	0.492	0.315	0.493	0.362	0.286	0.390	0.431
14	0.476	0.385	0.452	0.444	0.273	0.474	0.371	0.271	0.379	0.437
15	0.647	0.465	0.436	0.450	0.284	0.500	0.563	0.283	0.379	0.420
16	0.671	0.509	0.489	0.486	0.394	0.525	0.302	0.297	0.443	0.483
17	0.309	0.252	0.296	0.328	0.212	0.361	0.173	0.201	0.284	0.316
18	0.361	0.288	0.317	0.328	0.209	0.354	0.179	0.227	0.290	0.333
19	0.447	0.323	0.323	0.359	0.228	0.399	0.192	0.219	0.311	0.356
20	0.452	0.381	0.403	0.474	0.307	0.481	0.242	0.279	0.390	0.483
21	0.323	0.266	0.285	0.310	0.193	0.342	0.167	0.190	0.263	0.293
22	0.490	0.443	0.484	0.504	0.322	0.544	0.277	0.327	0.427	0.477
23	0.666	0.651	0.559	0.626	0.379	0.639	0.340	0.372	0.490	0.621
24	0.419	0.319	0.360	0.383	0.231	0.411	0.211	0.238	0.332	0.379
25	0.580	0.567	0.575	0.620	0.356	0.607	0.355	0.357	0.495	0.540
26	0.537	0.447	0.409	0.444	0.258	0.493	0.236	0.257	0.358	0.431
27	0.537	0.460	0.371	0.389	0.262	0.436	0.233	0.257	0.342	0.391

28	0.614	0.460	0.446	0.517	0.315	0.493	0.245	0.286	0.390	0.443
29	0.314	0.274	0.328	0.346	0.216	0.373	0.186	0.208	0.300	0.339
30	1.170	1.014	1.005	1.033	0.827	1.094	0.588	0.673	0.811	0.988
31	0.666	0.456	0.522	0.583	0.353	0.557	0.362	0.338	0.453	0.488
32	0.571	0.474	0.527	0.571	0.341	0.550	0.308	0.338	0.479	0.523
33	0.457	0.372	0.393	0.389	0.258	0.436	0.267	0.257	0.321	0.402
34	0.561	0.518	0.409	0.444	0.292	0.519	0.264	0.294	0.390	0.448
35	0.813	0.925	0.581	0.754	0.394	0.727	0.371	0.361	0.569	0.678
36	0.799	0.575	0.608	0.583	0.375	0.626	0.516	0.375	0.506	0.563
37	0.300	0.266	0.312	0.334	0.239	0.348	0.186	0.249	0.290	0.305
38	0.571	0.505	0.559	0.535	0.326	0.512	0.595	0.323	0.474	0.523
39	0.828	0.558	0.532	0.571	0.364	0.582	0.371	0.349	0.485	0.546
40	0.390	0.363	0.398	0.431	0.277	0.455	0.230	0.260	0.374	0.414
41	0.751	0.580	0.532	0.492	0.334	0.652	0.431	0.320	0.458	0.500
42	0.794	0.509	0.516	0.498	0.315	0.557	0.305	0.316	0.432	0.540
43	0.685	0.514	0.613	0.638	0.425	0.696	0.349	0.364	0.500	0.569
44	0.523	0.385	0.339	0.365	0.243	0.399	0.204	0.245	0.327	0.368
45	0.380	0.314	0.323	0.359	0.220	0.367	0.223	0.223	0.295	0.322
46	0.756	0.549	0.575	0.590	0.402	0.614	0.399	0.335	0.516	0.546
47	0.414	0.319	0.360	0.359	0.228	0.380	0.258	0.219	0.306	0.356
48	0.599	0.447	0.586	0.486	0.372	0.544	0.308	0.361	0.506	0.529
49	0.780	0.633	0.613	0.681	0.436	0.734	0.450	0.398	0.595	0.655
50	0.514	0.412	0.403	0.492	0.303	0.455	0.271	0.275	0.395	0.437
51	0.342	0.310	0.339	0.389	0.239	0.411	0.242	0.242	0.337	0.368
52	0.556	0.434	0.505	0.553	0.391	0.601	0.330	0.361	0.495	0.540
53	0.466	0.460	0.457	0.474	0.315	0.500	0.280	0.297	0.390	0.448
54	0.618	0.412	0.398	0.419	0.258	0.424	0.242	0.257	0.364	0.402
55	0.889	0.691	0.753	0.820	0.516	0.886	0.428	0.498	0.701	0.724
56	0.499	0.332	0.366	0.359	0.231	0.399	0.233	0.230	0.321	0.356
57	0.680	0.589	0.473	0.498	0.368	0.563	0.286	0.353	0.437	0.488
58	0.899	0.509	0.527	0.620	0.364	0.601	0.324	0.368	0.485	0.511

59	0.533	0.367	0.457	0.438	0.277	0.455	0.293	0.275	0.385	0.425
60	0.528	0.367	0.441	0.407	0.277	0.436	0.302	0.279	0.369	0.408
61	0.352	0.279	0.360	0.352	0.220	0.367	0.217	0.216	0.306	0.356
62	0.466	0.359	0.403	0.419	0.265	0.436	0.245	0.249	0.358	0.408
63	0.628	0.491	0.597	0.620	0.356	0.671	0.352	0.357	0.543	0.626
64	0.480	0.390	0.409	0.444	0.265	0.487	0.236	0.275	0.385	0.437
65	0.637	0.443	0.489	0.535	0.341	0.595	0.302	0.327	0.464	0.500
66	0.633	0.527	0.446	0.517	0.417	0.582	0.277	0.364	0.453	0.517
67	1.070	1.058	1.210	1.234	0.736	1.316	0.598	0.725	1.133	1.184
68	0.509	0.460	0.457	0.462	0.307	0.500	0.346	0.305	0.385	0.448
69	0.604	0.474	0.468	0.517	0.334	0.519	0.330	0.312	0.432	0.494
70	0.547	0.438	0.452	0.504	0.303	0.506	0.274	0.305	0.427	0.471
71	0.575	0.456	0.462	0.474	0.319	0.531	0.305	0.305	0.395	0.437
72	0.556	0.562	0.495	0.559	0.341	0.595	0.311	0.335	0.443	0.529
73	0.414	0.301	0.333	0.413	0.228	0.386	0.226	0.216	0.311	0.339
74	0.485	0.341	0.382	0.389	0.246	0.455	0.230	0.238	0.353	0.374
75	1.122	0.788	1.199	0.705	0.478	0.797	0.572	0.465	0.627	0.678
76	0.314	0.257	0.296	0.334	0.205	0.335	0.179	0.197	0.421	0.322
77	0.728	0.452	0.393	0.407	0.277	0.443	0.258	0.257	0.358	0.414
78	0.523	0.407	0.430	0.413	0.296	0.430	0.711	0.279	0.395	0.488
79	0.547	0.447	0.548	0.553	0.360	0.620	0.302	0.327	0.448	0.488
80	0.913	0.651	0.704	0.705	0.489	0.721	0.403	2.491	0.643	0.753
81	0.342	0.301	0.333	0.383	0.250	0.399	0.198	0.223	0.327	0.356
82	0.490	0.460	0.522	0.571	0.341	0.576	0.406	0.331	0.437	0.443
83	0.523	0.447	0.473	0.486	0.292	0.531	0.311	0.275	0.400	0.454
84	0.514	0.416	0.527	0.529	0.338	0.582	0.286	0.305	0.427	0.488
85	0.699	0.593	0.559	0.596	0.398	0.658	0.384	0.364	0.532	0.592
86	0.837	0.611	0.570	0.559	0.349	0.614	0.406	0.361	0.485	0.586
87	0.414	0.376	0.403	0.474	0.284	0.493	0.252	0.283	0.400	0.443
88	0.395	0.274	0.317	0.340	0.231	0.373	0.236	0.216	0.306	0.328
89	2.497	2.267	2.221	2.528	1.771	2.113	1.752	1.543	2.144	2.276

90	0.571	0.487	0.484	0.504	0.326	0.512	0.283	0.320	0.443	0.483
91	1.303	0.788	0.790	0.875	0.576	0.835	0.554	0.535	0.732	0.810
92	0.552	0.416	0.425	0.480	0.292	0.474	0.245	0.294	0.400	0.425
93	0.861	0.713	0.683	0.693	0.447	0.696	0.399	0.394	0.595	0.649
94	0.533	0.553	0.446	0.498	0.303	0.525	0.271	0.294	0.390	0.443
95	0.837	0.593	0.656	0.790	0.436	0.702	0.396	0.409	0.553	0.575
96	0.376	0.310	0.360	0.383	0.235	0.405	0.230	0.238	0.332	0.356
97	0.561	0.385	0.457	0.431	0.284	0.512	0.311	0.375	0.400	0.414
98	4.623	5.998	5.952	6.649	3.993	4.630	4.193	4.004	5.173	5.931
99	0.661	0.438	0.446	0.511	0.330	0.493	0.261	0.290	0.406	0.443
100	0.499	0.363	0.419	0.468	0.277	0.493	0.245	0.294	0.400	0.454
101	0.457	0.403	0.398	0.425	0.307	0.481	0.239	0.290	0.390	0.431
102	0.295	0.248	0.296	0.328	0.201	0.348	0.176	0.197	0.284	0.310
103	0.685	0.474	0.489	0.498	0.387	0.563	0.296	0.402	0.416	0.477
104	0.376	0.376	0.333	0.389	0.262	0.392	0.211	0.238	0.327	0.362
105	0.400	0.416	0.505	0.590	0.379	0.557	0.327	0.349	0.411	0.569
106	0.328	0.270	0.317	0.340	0.212	0.348	0.176	0.208	0.311	0.333
107	0.656	0.779	0.747	0.857	0.622	0.942	0.431	0.498	0.690	0.718
108	0.785	0.686	0.747	0.717	0.463	0.740	0.557	0.409	0.616	0.678
109	0.419	0.403	0.376	0.419	0.262	0.449	0.230	0.264	0.364	0.402
110	0.685	0.385	0.387	0.444	0.284	0.474	0.233	0.294	0.385	0.425
111	0.561	0.598	0.527	0.614	0.425	0.620	0.311	0.375	0.511	0.701
112	0.400	0.367	0.360	0.401	0.250	0.405	0.233	0.245	0.327	0.379
113	0.799	0.642	0.731	0.681	0.406	0.677	0.403	0.390	0.532	0.575
114	0.742	0.505	0.511	0.517	0.338	0.538	0.393	0.911	0.427	0.465
115	0.447	0.363	0.409	0.492	0.334	0.531	0.296	0.297	0.390	0.420
116	0.889	0.606	0.608	0.577	0.379	0.658	0.333	0.372	0.522	0.592
117	0.452	0.367	0.371	0.395	0.243	0.424	0.220	0.234	0.342	0.420
118	0.790	0.584	0.511	0.650	0.387	0.595	0.324	0.349	0.495	0.534
119	0.333	0.274	0.296	0.322	0.209	0.361	0.186	0.201	0.279	0.362
120	0.518	0.359	0.425	0.450	0.383	0.462	0.258	0.260	0.369	0.540

121	0.523	0.398	0.457	0.468	0.284	0.474	0.286	0.294	0.390	0.437
122	0.304	0.261	0.301	0.328	0.197	0.342	0.167	0.197	0.290	0.299
123	0.452	0.372	0.441	0.413	0.246	0.443	0.242	0.257	0.358	0.408
124	0.585	0.567	0.538	0.559	0.356	0.626	0.377	0.383	0.469	0.483
125	0.442	0.323	0.398	0.438	0.243	0.399	0.217	0.253	0.327	0.339
126	0.442	0.398	0.436	0.431	0.277	0.512	0.271	0.257	0.379	0.408
127	1.203	0.757	0.833	0.839	0.542	0.810	0.645	0.580	2.271	0.724
128	0.466	0.385	0.376	0.407	0.269	0.405	0.242	0.253	0.364	0.414
129	0.480	0.359	0.419	0.480	0.300	0.474	0.252	0.283	0.395	0.431
130	0.999	0.744	0.807	0.833	0.542	0.942	0.550	0.517	0.722	0.805
131	0.523	0.381	0.333	0.365	0.228	0.392	0.198	0.234	0.327	0.374
132	0.575	0.354	0.409	0.413	0.269	0.436	0.252	0.294	0.364	0.414
133	0.818	0.593	0.597	0.596	0.432	0.689	0.355	0.383	0.522	0.586
134	0.499	0.407	0.436	0.480	0.319	0.474	0.293	0.279	0.421	0.477
135	0.504	0.394	0.468	0.486	0.292	0.519	0.333	0.312	0.432	0.454
136	1.018	0.633	0.876	0.711	0.421	0.702	0.488	0.379	0.659	0.661
137	1.075	0.987	0.591	0.596	0.421	0.671	0.340	0.357	0.532	0.609
138	0.847	0.655	0.629	0.608	0.391	0.664	0.337	0.413	0.527	0.592
139	0.438	0.363	0.371	0.395	0.246	0.411	0.214	0.257	0.348	0.420
140	0.804	0.646	0.731	0.754	0.478	0.778	0.447	0.476	1.991	0.770
141	0.338	0.310	0.339	0.377	0.246	0.386	0.201	0.223	0.316	0.368
142	0.390	0.345	0.393	0.413	0.265	0.417	0.258	0.249	0.348	0.385
143	0.452	0.363	0.414	0.444	0.292	0.474	0.286	0.264	0.369	0.402
144	0.652	0.394	0.419	0.425	0.281	0.417	0.299	0.283	0.374	0.414
145	0.523	0.443	0.505	0.571	0.349	0.614	0.308	0.349	0.464	0.603
146	0.756	0.668	0.645	0.669	0.474	0.772	0.371	0.420	0.569	0.678
147	0.571	0.456	0.414	0.419	0.273	0.443	0.324	0.260	0.369	0.425
148	0.466	0.412	0.446	0.559	0.322	0.500	0.267	0.294	0.421	0.460
149	0.466	0.354	0.398	0.401	0.254	0.462	0.230	0.409	0.337	0.385
150	0.419	0.376	0.419	0.438	0.281	0.487	0.274	0.271	0.379	0.420

Table S2. Normalized IgG optical density values for Lyme disease positive (sera ID 1-48), negative (sera ID 49-78), and febrile patients (sera ID 79-150) from index test.

Sera ID	Borrelia spirochete species	Borrelia persistent forms	*Babesia microti*	*Bartonella henselae*	*Ehrlichia chaffeensis*	*Rickettsia akari*	Coxsackievirus	Epstein-Barr virus	Human parvovirus B19	Mycoplasma species
1	1.207	0.555	0.735	0.638	0.497	0.670	0.524	0.510	0.592	0.589
2	0.929	0.662	0.557	0.560	0.486	0.624	0.472	0.608	0.530	0.548
3	0.915	0.634	0.629	0.600	0.405	0.638	0.539	0.440	0.626	0.604
4	1.004	0.802	0.726	0.710	0.540	0.830	0.625	0.559	0.722	0.699
5	1.188	0.722	0.636	0.564	0.449	0.593	0.526	0.421	0.570	0.517
6	1.114	0.899	0.811	0.863	0.633	0.901	0.723	0.657	0.848	0.738
7	1.033	0.998	0.762	0.756	0.958	0.825	0.800	0.524	0.749	0.634
8	0.575	0.647	0.709	0.642	0.658	0.684	0.627	0.545	0.651	0.638
9	0.835	0.674	0.516	0.528	0.430	0.510	0.426	0.346	0.461	0.452
10	0.724	0.631	0.593	0.544	0.440	0.634	0.523	0.473	0.558	0.535
11	0.934	0.829	0.474	0.394	0.315	0.464	0.390	0.318	0.482	0.439
12	0.658	0.596	0.454	0.383	0.292	0.415	0.329	0.514	0.363	0.397
13	0.444	0.343	0.320	0.258	0.226	0.318	0.253	0.292	0.256	0.264
14	0.519	0.434	0.402	0.407	0.340	0.407	0.369	0.614	0.425	0.412
15	0.676	0.699	0.676	0.666	0.465	0.647	0.532	0.588	0.591	0.519
16	1.065	0.632	0.819	0.646	0.505	0.694	0.523	0.449	0.676	0.599
17	0.645	0.519	0.450	0.420	0.386	0.492	0.365	0.312	0.456	0.433
18	0.542	0.431	0.354	0.313	0.255	0.333	0.270	0.221	0.335	0.335
19	1.158	0.438	0.285	0.264	0.198	0.297	0.219	0.184	0.266	0.241
20	1.291	1.301	0.600	0.563	0.408	0.537	0.440	0.382	0.508	0.480
21	0.704	0.713	0.609	0.631	0.413	0.595	0.470	0.420	0.533	0.539
22	1.028	0.533	0.539	0.544	0.476	0.553	0.474	0.461	0.544	0.478
23	1.800	1.754	1.892	1.707	1.411	1.603	1.412	1.132	1.596	1.323
24	2.858	1.756	1.502	1.189	0.952	1.583	1.289	1.243	1.799	1.951
25	0.609	0.538	0.571	0.467	0.466	0.589	0.490	0.605	0.473	0.536
26	0.897	0.762	0.650	0.513	0.472	0.591	0.531	0.960	0.595	0.580
27	0.580	0.283	0.289	0.231	0.185	0.262	0.217	0.253	0.241	0.251

28	0.655	0.525	0.424	0.374	0.332	0.414	0.341	0.283	0.381	0.375
29	0.763	0.557	0.451	0.437	0.369	0.472	0.398	0.351	0.474	0.406
30	0.611	0.745	0.527	0.549	0.422	0.531	0.458	0.384	0.507	0.377
31	0.974	0.736	0.696	0.726	0.534	0.692	0.617	0.497	0.675	0.668
32	1.449	0.903	0.850	0.812	0.606	0.791	0.750	0.567	0.792	0.764
33	0.914	0.558	0.488	0.450	0.359	0.512	0.434	0.764	0.461	0.450
34	0.761	0.701	0.572	0.542	0.412	0.578	0.519	0.430	0.546	0.532
35	1.394	0.799	0.845	0.926	0.714	0.818	0.734	0.761	0.821	0.715
36	0.997	0.654	0.656	0.608	0.501	0.690	0.599	0.566	0.657	0.559
37	0.628	0.594	0.673	0.541	0.543	0.601	0.525	0.642	0.585	0.468
38	0.611	0.615	0.706	0.680	0.533	0.632	0.575	0.916	0.596	0.535
39	0.785	0.658	0.610	0.635	0.462	0.615	0.545	0.500	0.604	0.505
40	1.174	0.852	0.664	0.607	0.501	0.619	0.533	0.445	0.610	0.624
41	0.432	0.411	0.416	0.371	0.308	0.392	0.334	0.382	0.375	0.350
42	0.757	0.699	0.624	0.627	0.476	0.641	0.545	0.522	0.603	0.555
43	0.761	0.724	0.552	0.568	0.455	0.528	0.460	0.376	0.544	0.465
44	0.700	0.626	0.564	0.518	0.444	0.557	0.457	0.425	0.480	0.484
45	0.313	0.303	0.288	0.308	0.271	0.359	0.293	0.221	0.305	0.264
46	1.131	1.150	1.187	1.125	0.859	1.076	0.929	0.922	0.950	0.882
47	1.001	0.950	0.886	0.964	0.684	0.912	0.790	0.781	0.793	0.695
48	0.745	0.743	0.620	0.607	0.478	0.595	0.613	0.455	0.500	0.510
49	0.950	1.018	0.784	0.720	0.642	0.781	0.740	0.548	0.690	0.641
50	0.548	0.528	0.482	0.580	0.390	0.495	0.392	0.332	0.441	0.362
51	0.691	0.717	0.836	0.664	0.667	0.806	0.767	0.614	0.714	0.613
52	0.966	0.893	0.949	0.836	0.770	0.886	0.759	0.562	0.758	0.649
53	0.456	0.526	0.528	0.534	0.465	0.531	0.479	0.382	0.504	0.434
54	0.982	0.542	0.540	0.563	0.419	0.557	0.454	0.418	0.535	0.505
55	0.623	0.479	0.497	0.471	0.396	0.525	0.426	0.373	0.474	0.470
56	1.108	0.438	0.392	0.381	0.312	0.436	0.371	0.409	0.395	0.381
57	1.054	0.521	0.580	0.498	0.399	0.578	0.474	0.575	0.504	0.490
58	0.862	0.624	0.473	0.406	0.362	0.461	0.422	0.982	0.418	0.417

59	0.599	0.295	0.274	0.304	0.221	0.287	0.242	0.197	0.285	0.264
60	0.566	0.347	0.304	0.306	0.217	0.305	0.253	0.211	0.276	0.251
61	1.247	1.089	0.921	0.943	0.798	0.918	0.812	0.617	0.880	0.817
62	1.181	0.698	0.688	0.691	0.571	0.777	0.613	0.555	0.650	0.588
63	0.865	0.657	0.647	0.553	0.481	0.592	0.501	0.432	0.549	0.457
64	0.959	0.927	0.837	0.824	0.694	0.909	0.755	0.635	0.817	0.684
65	1.077	0.680	0.692	0.729	0.628	0.667	0.552	0.537	0.629	0.528
66	0.762	0.732	0.670	0.562	0.495	0.587	0.488	0.488	0.506	0.508
67	2.247	1.941	2.194	2.036	1.432	2.159	1.465	1.383	1.798	1.719
68	1.359	1.148	1.084	1.131	0.870	1.078	0.916	0.861	0.988	0.891
69	0.818	0.780	0.705	0.737	0.625	0.708	0.776	0.522	0.712	0.634
70	0.568	0.442	0.482	0.525	0.409	0.554	0.446	0.367	0.474	0.428
71	0.979	0.569	0.596	0.530	0.442	0.593	0.490	0.613	0.524	0.478
72	0.787	0.523	0.456	0.468	0.441	0.482	0.429	0.355	0.463	0.412
73	0.681	0.588	0.485	0.559	0.401	0.514	0.401	0.333	0.454	0.401
74	0.592	0.555	0.541	0.511	0.466	0.543	0.494	0.667	0.516	0.439
75	0.876	0.634	0.724	0.663	0.578	0.696	0.602	0.599	0.643	0.570
76	0.617	0.548	0.558	0.583	0.504	0.560	0.486	0.400	0.553	0.510
77	0.841	0.694	0.741	0.604	0.570	0.724	0.646	0.542	0.652	0.604
78	0.860	0.660	0.638	0.626	0.545	0.672	0.613	0.541	0.636	0.609
79	0.690	0.590	0.608	0.600	0.482	0.621	0.522	0.582	0.535	0.557
80	1.131	0.870	0.888	0.737	0.707	0.776	0.722	0.793	0.748	0.855
81	0.785	0.679	0.570	0.657	0.578	0.591	0.541	0.468	0.600	0.614
82	0.545	0.635	0.577	0.594	0.506	0.564	0.520	0.419	0.523	0.484
83	0.679	0.669	0.737	0.671	0.568	0.599	0.589	0.539	0.514	0.473
84	0.587	0.590	0.457	0.489	0.421	0.528	0.447	0.346	0.458	0.395
85	1.186	1.077	1.144	1.121	1.058	1.202	0.953	0.799	0.858	0.974
86	0.720	0.673	0.671	0.616	0.554	0.647	0.554	0.412	0.565	0.539
87	0.601	0.570	0.481	0.500	0.387	0.558	0.419	0.321	0.473	0.428
88	0.533	0.503	0.565	0.597	0.447	0.532	0.450	0.408	0.418	0.427
89	1.479	1.583	1.819	1.710	1.225	1.682	1.481	1.243	1.494	1.378

90	0.985	0.438	0.565	0.512	0.533	0.568	0.544	0.370	0.514	0.426
91	0.966	0.924	0.881	0.813	0.780	0.820	1.045	0.587	0.813	0.728
92	0.751	0.647	0.648	0.616	0.482	0.680	0.539	0.469	0.560	0.576
93	0.695	0.626	0.597	0.520	0.376	0.536	0.425	0.395	0.509	0.455
94	1.057	0.822	0.669	0.634	0.507	0.606	0.513	0.698	0.589	0.570
95	0.845	0.720	0.615	0.735	0.504	0.569	0.502	0.577	0.543	0.490
96	0.587	0.588	0.631	0.539	0.571	0.567	0.526	0.467	0.483	0.504
97	0.550	0.498	0.474	0.510	0.448	0.581	0.462	0.401	0.479	0.454
98	1.429	1.454	1.621	1.592	1.165	1.554	1.492	1.306	1.404	1.160
99	0.425	0.394	0.425	0.366	0.287	0.381	0.350	0.261	0.350	0.339
100	0.540	0.516	0.427	0.391	0.364	0.433	0.352	0.420	0.424	0.397
101	0.582	0.484	0.470	0.472	0.346	0.491	0.444	0.375	0.503	0.480
102	0.584	0.536	0.461	0.454	0.363	0.457	0.406	0.409	0.439	0.437
103	0.597	0.503	0.355	0.353	0.285	0.361	0.311	0.305	0.335	0.310
104	0.370	0.347	0.332	0.347	0.243	0.318	0.252	0.237	0.309	0.299
105	0.814	0.675	0.646	0.650	0.572	0.670	0.596	0.451	0.639	0.632
106	0.598	0.476	0.496	0.497	0.420	0.485	0.419	0.366	0.447	0.437
107	0.537	0.498	0.474	0.428	0.357	0.478	0.411	0.335	0.452	0.420
108	0.425	0.394	0.376	0.343	0.293	0.374	0.349	0.341	0.345	0.334
109	0.501	0.371	0.404	0.347	0.269	0.361	0.321	0.255	0.325	0.337
110	1.005	0.721	0.653	0.589	0.498	0.615	0.528	0.512	0.508	0.499
111	0.742	0.698	0.719	0.683	0.551	0.643	0.583	0.629	0.636	0.559
112	0.454	0.443	0.390	0.387	0.309	0.359	0.307	0.263	0.297	0.279
113	1.059	0.839	0.669	0.650	0.572	0.742	0.588	1.313	0.603	0.565
114	0.756	0.573	0.601	0.608	0.530	0.598	0.520	0.444	0.557	0.502
115	0.880	0.489	0.486	0.429	0.437	0.505	0.444	0.372	0.439	0.411
116	1.078	0.456	0.347	0.306	0.273	0.379	0.320	0.249	0.342	0.299
117	0.714	0.580	0.579	0.512	0.406	0.593	0.486	0.494	0.439	0.513
118	1.455	1.351	0.707	0.615	0.509	0.702	0.588	0.490	0.563	0.538
119	1.720	1.039	0.899	0.787	0.531	0.958	0.693	0.627	1.125	1.159
120	0.607	0.586	0.564	0.597	0.426	0.587	0.454	0.587	0.476	0.410

121	0.725	0.528	0.684	0.588	0.463	0.588	0.460	0.376	0.520	0.570
122	0.714	0.726	0.503	0.491	0.415	0.540	0.414	0.356	0.512	0.479
123	0.602	0.509	0.484	0.424	0.366	0.471	0.432	0.298	0.468	0.439
124	0.399	0.347	0.338	0.322	0.253	0.350	0.275	0.226	0.315	0.289
125	0.525	0.477	0.464	0.429	0.361	0.422	0.465	0.288	0.411	0.372
126	0.563	0.544	0.474	0.493	0.392	0.436	0.378	0.423	0.441	0.424
127	0.804	0.724	0.736	0.736	0.726	0.703	0.647	0.517	0.714	0.630
128	0.825	0.589	0.633	0.595	0.483	0.585	0.525	0.470	0.500	0.470
129	1.066	0.803	0.843	0.792	0.664	0.738	0.651	0.895	0.662	0.702
130	0.717	0.543	0.539	0.474	0.465	0.535	0.429	0.367	0.433	0.410
131	0.714	0.636	0.621	0.585	0.436	0.630	0.520	0.523	0.545	0.532
132	0.890	0.397	0.389	0.373	0.288	0.401	0.351	0.285	0.341	0.351
133	0.661	0.490	0.497	0.431	0.334	0.491	0.402	0.315	0.395	0.398
134	0.649	0.481	0.403	0.419	0.327	0.440	0.384	0.294	0.424	0.371
135	0.554	0.618	0.513	0.513	0.412	0.509	0.447	0.410	0.474	0.430
136	1.125	0.566	0.485	0.508	0.422	0.526	0.408	0.345	0.471	0.441
137	0.484	0.418	0.460	0.437	0.382	0.500	0.422	0.341	0.424	0.415
138	0.850	0.559	0.431	0.376	0.306	0.440	0.370	0.395	0.398	0.375
139	0.859	0.659	0.704	0.532	0.489	0.573	0.623	0.518	0.611	0.548
140	0.971	0.699	0.606	0.564	0.480	0.602	0.546	0.409	0.520	0.540
141	0.670	0.553	0.469	0.509	0.425	0.445	0.418	0.409	0.474	0.478
142	0.659	0.557	0.511	0.518	0.363	0.466	0.362	0.374	0.434	0.407
143	0.890	0.894	0.672	0.596	0.574	0.631	0.581	0.799	0.604	0.564
144	0.873	0.367	0.383	0.358	0.292	0.360	0.341	0.270	0.339	0.329
145	0.436	0.373	0.354	0.354	0.258	0.364	0.299	0.820	0.347	0.315
146	1.724	1.569	1.536	1.475	1.050	1.626	1.320	1.135	1.450	1.333
147	0.688	0.535	0.455	0.411	0.326	0.445	0.406	0.380	0.427	0.430
148	1.140	0.830	0.907	0.897	0.638	0.901	0.670	0.612	0.809	0.661
149	1.660	0.965	0.861	0.737	0.589	0.747	0.671	0.486	0.741	0.733
150	0.721	0.665	0.679	0.590	0.492	0.674	0.549	0.500	0.843	0.495
